# Supplementary material for: A Contrast-Enhanced CT-Based Deep Learning System for Preoperative Prediction of Colorectal Cancer Staging and RAS Mutation
Source: Cancers (Basel). 2023 Sep 10;15(18):4497. doi: 10.3390/cancers15184497 (PMC10526233; doi:10.3390/cancers15184497)
Supplement: Supplementary file 1 [file cancers-15-04497-s001.zip › cancers-2585781-supplementary.pdf]

Supplementary materials

RAS mutation analysis

DNA was extracted from formalin-fixed paraffin-embedded (FFPE) tumour sections using the QIAamp DNA FFPE Tissue Kit (Qiagen). Mutations of KRAS (exons 2, 3, and 4), NRAS (exons 2, 3, and 4), and BRAF (V600E) were analysed by a next-generation sequencing (NGS) method.
